# Clinic of Dr. Pancoast

**Published:** 1843-12-23

**Authors:** H. T. Child


### PROFESSOR PANCOAST CASE OF PARACENTESIS CEREBRI,

*At the Clinic of Jefferson Medical College, November 15th, 1843.*

(Reported by H. T. Child.)

Prof. Pancoast introduced before the class a child, æt. 7 months, with enormous enlargement of the head, the consequence of chronichy drocephalus. This child, the lecturer observed, is of German descent, and I am informed was active and lively at birth, but, unusually dark coloured, the blueness gradually diminishing so as to disappear altogether by the fourth or fifth day. At the end of five weeks the child was seized with convulsions without apparent cause, which recurred at intervals of a few minutes for several days. The case was judiciously managed by Dr. Bournonville of this city. The child recovered so as to be able to take its nourishment well and seemed to thrive; some permanent affection of the brain soon after began to manifest itself, by occasional squinting, and an almost constant rolling motion of the head, which began gradually to increase in size. The first sign of the increase of the head, was apparent at the anterior fontanelle, which was enlarged by recession outwards and backwards of the parietal bones, and gave upon pressure a sense of fluctuation. It is about two weeks since I first saw the patient; the head then measured twenty-one inches around above the ears, nineteen and a half from the forehead to the nape of the neck. The anterior fontanelle was enormously enlarged and of a lozenge shape, running from before backwards along the sagittal suture, from the frontal to the occipital bone, a distance of seven and a half inches, and from side to side along the coronal suture seven inches. The sutures at the anterior side of the squamous portion of the temporal bones, were also partly opened; all the others were unusually firmly ossified, in consequence apparently of the bones being pressed together at the posterior and lower part of the cranium, by the distension which had opened the fontanelle. The child squinted constantly with the left eye, and was distorted on the right side of the back by the contraction of the spinal muscles. I tapped the brain with a small gold trochar, in the presence of several medical gentlemen, and removed a gill of transparent fluid which was saline to the taste, and on being analyzed by Mr. Frederick L. Johns, of this city, was found to contain in

| | | |
|---|---|---|
| 1000 parts | 4 parts | of carbonate of soda. |
| | 13 " | of muriate of soda. |
| Specific Gravity 1.035 | 3 " | animal mucilage and extractive matter. |
| | 886 " | water. |

The child, a few moments before, had been in convulsions; it seemed relieved by the operation and took the breast immediately. The flap sunk down after the operation so as to bring out the bony edges of the fontanelle in relief, but the bones of the cranium were too firmly ossified toward the base to be made to approach each other, presenting as it appears to me an almost insurmountable obstacle in the way of cure. The flap of the scalp has again raised, as you observe, by a reaccumulation of the fluid within, though the head is found to be diminished nearly an inch in circumference.

Tapping offers the only prospect of relief in these cases, and that I believe is slight. The parents are apprised of this, and are even prepared for a fatal result, in case it should follow the evacuation of the fluid. I do not, however, believe it likely to happen, and I intend to guard against it as much as possible by taking away but a moderate quantity of fluid at one time, and repeating the operation from time to time, rather than take away at once the great amount that must necessarily be collected to have formed this great expansion of the cranium.

This operation has been performed many times: by Messrs. Conquest, Russel and Butcher of Great Britian, Graefe of Germany, and Malgaigne, of Paris, and several times in this country. According to Mr. Conquest's statement he has been successful in curing ten cases out of nineteen. There has been some difference of opinion as to the most appropriate place for the puncture. Mr. Conquest prefering the track of the frontal suture. Mr. Russel, one of the sides of the fontanelle. I have selected the middle part of the lateral wing of the fontanelle, as the point most likely to reach the cavity of the lateral ventricle, and avoid the track of the middle artery of the dura mater, and will operate on the right side as this is the most prominent. The trochar must pass through the scalp, the three membranes of the brain, and through the thinned and expanded portion of the brain above the ventricles. The fluid must have collected in the ventricles, and expanded the brain, so as to have unfolded the convolutions, breaking down the partition between the ventricles.

The trochar was then inserted without any manifestation of uneasiness on the part of the child, and about three ounces and a half of fluid removed; the flap of skin sunk down, as in the former operation. Before removing the trochar a plug was inserted in the orifice, lest the suction made in withdrawing it should draw some air into the cavity of the ventricle.

The child gaped and seemed like a person just wakening from a deep sleep.

